# Analysis of sperm chromatin packaging and reproductive biomarker to evaluate the consequence of advanced male age

**DOI:** 10.3389/fendo.2023.1092603

**Published:** 2023-04-14

**Authors:** Riffat Bibi, Sarwat Jahan, Salma Kafeel Qureshi, Suhail Razak, Tayyaba Afsar, Ali Almajwal, Mashal Kafeel Qureshi, Mohammad Eid Hammadeh, Houda Amor

**Affiliations:** ^1^ Department of Animal Sciences, Faculty of Biological Sciences, Quaid-i-Azam University Islamabad, Islamabad, Pakistan; ^2^ Department of Reproductive Health Sciences, Salma and Kafeel Medical Centre, Islamabad, Pakistan; ^3^ Department of Community Health Sciences, College of Applied Medical Sciences, King Saud University, Riyadh, Saudi Arabia; ^4^ Department of Obstetrics, Gynecology and Reproductive Medicine, Saarland University Clinic, Homburg, Germany

**Keywords:** sperm chromatin integrity, assisted reproductive procedures, sperm deoxyribose nucleic acid fragmentation index, reproductive marker, male age

## Abstract

In this study, the semen parameters, sperm chromatin integrity, antioxidant enzyme levels, and reproductive hormone levels of subfertile male subjects from Pakistan were assessed in relation to their age. Data on the demographic characteristics of the 750 study participants, including their general health, body mass index (BMI), and reproductive status, were collected from subfertile men from Pakistan. Semen and blood were collected to determine standard semen parameters, sperm chromatin dispersion (Halosperm-SCD), sperm chromatin integrity using toluidine blue (TB) staining, sperm chromatin maturity using chromomycin A3 (CMA3+) staining, and reproductive hormone (FSH, LH, prolactin and testosterone levels). The patients were divided into three groups according to their age: Group 1 included male subjects aged 30 years or less (*n* = 90), Group 2 included male subjects between the ages of 31 and 40 years (*n* = 330), and Group 3 included male subjects over 40 years of age (*n* = 330). Conventional semen parameters, reactive oxygen species (ROS), superoxide dismutase (SOD), guaiacol peroxidase (GPX), catalase (CAT), and lipid peroxidation (MDA) did not statistically (*p* > 0.05) differ with increasing male age or between different age groups. When compared to younger men (<30 years), sperm SCD (23.2 ± 0.88%) was significantly (*p* = 0.01) lower as compared to male patients aged >40 years (26.6 ± 0.6%). The concentration of LH, FSH, and testosterone levels were comparable between the groups (*p* > 0.05), while a significant (*p* = 0.04) increase in sperm chromatin immaturity CMA3+ (30 ± 0.71%) was observed in the old age group (>40 years) compared to the <30-year group (26.6 ± 1.03%). A positive association was observed between advanced male age and sperm chromatin dispersion (SCD) (*r* = 0.124, *p* = 0.001) and decondensation (CMA3+) (*r* = 0.1, *p* = 0.009). Despite potential limitations, this study has been carried out with extensive information on the potential risk of male age on sperm integrity. The present study demonstrated the impact of male age on male reproductive health, as these patients had a higher percentage of sperm chromatin damage (SCD) in their semen. Sperm DNA damage assessment will help in the evaluation and diagnosis of the underlying cause of poor fertility and can help clinicians in selecting the right treatment options. Male age is one of the factors that have an impact on the decline in male fertility. As a result, it is preferable for patients receiving assisted reproductive technology to be younger.

## 1 Introduction

The risk of infertility and poor child health increases with delayed family planning and older parents. While the effects of aging on oogenesis have been extensively studied, spermatogenesis has received less attention (1). It is estimated that the prevalence of male subfertility between the ages of 15 and 50 years is up to 6%. Approximately 25% of couples experience male factor subfertility (2, 3). It has been reported that in male partners opting for semen analysis, over 50% of men presented with abnormal semen parameters. In recent years, advancing age becomes a key factor contributing to debility in reproductive health indices in both sexes. Old male patients have augmented estrogen levels, due to the amplification of aromatase; through a negative feedback loop, men display indications of hypogonadotropic hypogonadism. These hormonal fluctuations, besides augmented oxidative stress, lipotoxicity, and instabilities in the absorptions of adipokines, directly distress the gonads, peripheral reproductive organs, and the embryo (4). It is generally well accepted that reproductive function highly correlates with the degree of adiposity, nutrition, or metabolic condition related to food intake in human medicine (5, 6). Male age >40 years is associated with reduced semen quality. Furthermore, infection, immunological factors, trauma, or surgical insult to the male reproductive organs, and exposure to toxic chemicals or other materials are all known acquired factors that contribute to male subfertility (2, 7, 8). Similarly, a direct association was found between men’s age and semen quality even after adjustment for reproductive hormones (9).

Semen analysis is a routine and simple method for assessing male fertility status. However, alone, it is not sufficient to predict assisted reproductive outcomes (10, 11). With the development of new predictive tools to identify male fertility potential, the sperm deoxyribonucleic acid fragmentation index is a commonly used technique involving different methods (10, 12). For identification of the DNA fragmentation index, we used SCD assay (13–15). Chromomycin A3 (CMA3) has been used for the evaluation of sperm chromatin condensation, which is indirectly associated with its integrity since this fluorochrome binds to the guanine–cytosine dinucleotide region of DNA competitively with protamines that bind to the same region (16). CMA3 has been used as an indirect measure of the protamination state of nuclear chromatin. On the other hand, several authors affirm that the presence of protamine-deficient spermatozoa CMA3+ is associated with DNA integrity (17, 18). They base it on the fact that protamines are nuclear proteins that play a key role in the integrity of sperm DNA since they are responsible for the integrity stability and packaging of sperm DNA until the paternal genome is introduced into the oocyte during fertilization.

The relationships between age, semen characteristics, male reproductive hormones, sperm DNA fragmentation, chromatin structure, and ART outcome have been inconsistently correlated, according to numerous studies and meta-analyses (1, 19, 20). Giving birth at an appropriate male age can reduce the risk of disease in future generations. Regarding IVF and/or ICSI, despite the fact that numerous clinical studies have been carried out to evaluate the negative effects of human sperm DNA damage on reproductive outcomes, the findings from these studies are still debatable. Some researchers claim that sperm DNA damage has no negative effects on the rate of fertilization and pregnancy rate (21–23), while others claim that there is a link between DNA fragmentation and decreased fertility and pregnancy outcome (2, 24, 25). Moreover, other factors such as age would be the leading cause of lower pregnancy rates and failure of reproductive outcomes. Therefore, the overall health and normal age of parents should be considered in couples as an important concern in attaining successful reproductive outcomes. We aimed to investigate the correlation of male age on semen parameters (concentration, motility, morphology, and vitality), oxidative stress, hormonal levels, SCD, and chromatin compaction markers.

## 2 Materials and methods

### 2.1 Study design and ethical clearance

The research was conducted at the Faculty of Biological Sciences, Reproductive Physiology Laboratory, Department of Zoology, and Quaid-i-Azam University-Islamabad Pakistan. All study participants provided consent and signed informed consent forms. The criteria for participation in the study were that the couples give their informed written consent. The ethical approval to conduct this study was obtained from the Ethics Committee of Salma Kafeel Medical Centre Islamabad Pakistan No, SKMC&FGS-010-2016. and the Bio-Ethic committee of the Department of Zoology, Quaid-i-Azam University, and Islamabad # BEC-FBS-QAU2016-77.

### 2.2 Participants

Inclusion and exclusion criteria were as follows: couples undergoing their first ovarian stimulation (who remained unsuccessful in achieving pregnancy after trying for 12 or more months, with male partner age range between 20 and 49 years from January 2016 to October 2021); patients with recent fever, abnormalities of the external genitalia, abnormal karyotyping, cryptorchidism, varicoceles, presence of anti-sperm antibodies, azoospermia, or severe oligoasthenoteratozoospermia; those taking treatment that can alter spermatogenesis; patients with chronic diseases (e.g., liver/renal disease, patients with hypertension, diabetes, and andrological disorders); and those with an identified subfertility factor in the female partner were not included. All patients were properly advised of the associated risks of IVF therapy and completed an informed permission form to allow researchers to utilize their clinical data. The patients were divided into three groups according to their age: Group 1 included male patients aged 30 years or less (*n* = 90) (the data obtained from male patients aged less than 30 years compared to other groups were lesser in record and fewer responders were available), Group 2 included male subjects between the ages of 31 and 40 years (*n* = 330), and Group 3 included male subjects over 40 years of age (*n* = 330). The study protocol was developed following the Declaration of Helsinki (26). The sample size was calculated using the formula used before (27, 28).

### 2.3 Sampling technique and data collection

Data collection was done through face-to-face interviews and electronically and the following characteristics of the couple were documented and evaluated: age (full years), duration of subfertility (years), history of hypertension or diabetes mellitus, family history, obesity, subfertility, and genetic disease during the first visit by an informal interview with the couple. The research committee of the Quaid I Azam University in Islamabad’s Department of Reproductive Physiology examined and approved the study protocol and questionnaire. The survey responses were kept private. The data collector and skilled medical personnel entered the information into a database. The data collector made sure that the interviews and data were kept private. The lead investigator was the only person with access to the complete collection of data. Before data and sample collection, couples were assured that their identity would be kept anonymous.

### 2.4 Body mass index

All couples’ height and weight were measured by a skilled nurse at the initial visit. Weight divided by height squared was used to compute BMI according to the classification standards of the global organization.

### 2.5 Outcomes

Semen sample volume, concentration, motility, and morphology were evaluated. SCD assay, chromatin integrity using toluidine blue (TB) staining, and CMA3 staining have been used as an indirect measure of the protamination state of nuclear chromatin and chromatin integrity.

### 2.6 Semen standard parameter analysis

After masturbation, the semen sample was collected, after 2–5 days of abstinence, and the semen sample was analyzed after 30 min of liquefaction at 37°C. Each sample was subjected to analysis for seminal characteristics. Semen parameters were assessed according to WHO 2010 standards; to summarize, sperm number was determined, the sperm motility was determined using a Leica microscope DM300 scoring at least 100 spermatozoa/slide, and morphology was determined using Diff-Quik staining. Sperm deformity index (SDI) and Teratozoospermic index (TZI) are calculated as described by Cooper et al. (29).

According to Jeyendran et al., the hypo-osmotic swelling test (HOS-test) was used for the assessment of membrane integrity of spermatozoa. A 100-µl sample of sperm suspension was added to 1 ml of hypoosmotic solution (equal parts of 150 mOsmol fructose and 150 mOsmol sodium citrate solutions), followed by 60 min of incubation at 37°C. After incubation, a minimum of 200 spermatozoa were examined per slide under a light microscope and the percentage of spermatozoa that showed typical tail abnormalities (curly tail) indicative of swelling were calculated (30).

### 2.7 Biochemical studies

While oxidant concentration of the ROS assessment method was previously published in detail, semen samples were examined to test antioxidant enzyme levels including superoxide dismutase (SOD) (units/mg of protein) (31), guaiacol peroxidase (GPX), catalase (CAT) (32), and lipid peroxidation *via* malondialdehyde (MDA) (33) on a UV spectrophotometer (Agilent 8453). ROS were estimated using the protocol of Novotný et al. (34); briefly, the liquefied semen was centrifuged at 300*g* for 7 min, seminal plasma was removed, and the pellet of cells was washed in PBS (isotonic solution, pH = 7.4) and spun again and decanted. Washed cells were suspended in PBS to adjust sperm concentration to 1.25 × 10^6^/ml. ROS production was measured after the addition of 10 μl of 5 mM freshly prepared solution of luminol (5-amino-2,3-dihydro-1,4-phthalazinedione, Sigma Chemical Co., St. Louis, MO, USA) in dimethyl sulfoxide (DMSO, Sigma Chemical Co.) to 400 μl of spermatozoa suspension. A tube containing 400 μl of PBS and 10 μl of luminol solution served as a blank. Chemiluminescence was measured integrally for 15 min using the Digene DCR-1 single detector luminometer (Digene Diagnostics, Inc., Gaithersburg, MD, USA). Results were expressed in relative light units (RLU) per minute and 20 × 10^6^ spermatozoa.

The other semen fraction was tested for sperm DNA fragmentation (SCD), and chromatin maturity (CMA3+, TB+) was evaluated.

### 2.8 Sperm chromatin dispersion assay

As previously reported, the SCD test was conducted using a Sperm Nucleus DNA Integrity Kit (SCD) from Shenzhen Huakang Biomed Co., Ltd., Shenzhen, China (35). The technique that was carried out was as follows: A tube containing fluidized agarose received 60 μl of semen sample before being dropped onto a glass slide and covered with a glass coverslip. After 4 min at 4°C, the coverslip was removed. Following acid denaturation for 7 min, lysis for 20 min was performed. The slide was then thoroughly cleaned for 3 min with plenty of distilled water before being dehydrated for 2 min in successive ethanol washes of 70%, 90%, and 100%. Wright’s staining was followed by the manual counting of 500 spermatozoa per slide to assess the integrity of the sperm DNA under bright-field microscopy. To assess the level of sperm DNA integrity, the dispersion of sperm DNA was calculated. If the value of SCD was found to be less than 30%, it was considered to be normal (36).

### 2.9 Toluidine blue staining

TB was used to measure chromatin integrity (37). Spermatozoa’s two smears were fixed with freshly prepared 96% ethanol and acetone (1:1), and the slides were treated with 0.1 M HCl at 4°C for 5 min and then rinsed three times with distilled water for 2 min each. After 5–10 min, the slides were rinsed with distilled water and coated with TB solution (0.05% TB in 50% McIlvain citrate phosphate buffer, pH 3.5–4). The slides were dehydrated in ethanol baths one after the other (70%, 96%, and 100%). Finally, per sample, 200 spermatozoa were counted under an optical microscope after the slides were coated and mounted with xylene at room temperature (2–3 min). A cationic dye is TB. It can attach to DNA with damaged or loosely packed phosphate residues that are negatively charged. The cells were divided into two groups: light blue cells (TB− cells; normal chromatin structure) and dark violet cells (TB+ cells; aberrant chromatin structure).

### 2.10 Chromomycin A3 staining

Semen smear slides were settled in a 3:1 solution of methanol and glacial acetic acid at 4°C for 20 min before actually air-drying at room temperature for 20 min. A 100-L CMA3 solution was added to the slides for 20 min (38). The CMA3 solution was composed of 0.25 mg/ml CMA3 in McIlvain’s buffer (pH 7.0) with 10 mmol/L MgCl_2_. The films were washed in a buffer before getting mounted in a 1:1 v/v PBS-glycerol solution. After that, these same slides were kept at 4°C for 24 h. A fluorescent microscope was used to assess luminescence. On every slide, 200 sperm cells are assessed at probability sampling. CMA3 immunofluorescence was tested by separating sperm cells that stain bright yellow (CMA3+) versus those that light-color a dull yellow (CMA3−).

### 2.11 Statistical analysis

Data were methodically imported to Microsoft Excel 2010 from the medical record and the interviewer. The Statistical Package for Social Sciences (SPSS) 20 IBM program was used for all statistical studies (Armonk, NY). Data were presented as mean ± SD. To compare the percentage, the ANOVA with Tukey’s test was chosen for the statistical analysis. Age-based groupings of the male subjects recruited for the current study were created. Age was the independent variable, while sperm DNA damage, chromatin maturity parameter, ROS, and semen parameter were considered dependent variables and values were compared to male BMI. Pearson correlation analysis was performed between the various parameters. Simple linear regression analysis was conducted to identify the relationship between male age as an independent variable with dependent variables including CMA3+, SCD, ROS, and TMS. The Hosmer–Lemeshow goodness of fit test was used to determine the model’s dependability. A *p-*value of <0.05 was considered to be statistically significant.

## 3 Results

### 3.1 Demographic parameters

The mean demographic parameters, including age (years), BMI (kg/m^2^), and fertility duration (years) evaluated in 750 couples enrolled in this study, are reported in **Table 1**.

**Table 1:** Demographic characteristics of couples included in the study.

	<30 years (*n* = 90)	30–40 years (*n* = 330)	>40 years (*n* = 330)	Total (*n* = 750)
Male age (years)	28.06 ± 0.30	36.24 ± 0.18	45.40 ± 0.32	38.80 ± 0.35
Male BMI (kg/m^2^)	22.79 ± 0.23	23.03 ± 0.11	22.71 ± 0.15	22.89 ± 0.08
Female age (years)	27.46 ± 0.66	32.35 ± 0.35	35.62 ± 0.45	32.79 ± 0.28
Female BMI (kg/m^2^)	26.89 ± 0.40	27.16 ± 0.19	26.75 ± 0.26	26.99 ± 0.14
Infertility duration (years)	5.03 ± 0.36	8.40 ± 0.31*	12.50 ± 0.55**	9.29 ± 0.28

Values represent mean ± SEM; BMI, body mass index, n = number of patients.

*p < 0.05, **p < 0.01.

### 3.2 Semen standard parameters, and biochemical and hormonal analysis

The mean conventional semen parameters, including concentration, normal morphology, total motile sperms (TMS %), HOS %, ROS (U/min), GPX (nmol), SOD (U/min), MDA (nmol/ml), and hormonal levels [FSH (mIU/ml), LH (mIU/ml), prolactin (mIU/ml), and testosterone (ng/ml) levels], were comparable in all age groups (**Table 2**).

**Table 2:** The effects of male age on semen parameters, biochemical profile, and reproductive hormone concentration in studied groups.

	Below 30 years (*n* = 90)	30 to 40 years (*n* = 330)	Above 40 years (*n* = 330)	Total (*n* = 750)
Semen parameters				
Semen volume (ml)	4.03 ± 0.18	3.74 ± 0.09	3.93 ± 0.14	3.85 ± 0.07
pH	8 ± 0.00	8.00 ± 0.01	8.15 ± 0.15	8.05 ± 0.05
Liquefaction time (min)	31.85 ± 0.93	33.36 ± 0.97	31.07 ± 0.39	32.43 ± 0.56
WBC/HPF	3.32 ± 0.26	3.01 ± 0.13	3.02 ± 0.18	3.06 ± 0.10
Concentration ×10^6^/ml	62.02 ± 8.13	56.97 ± 3.29	53.32 ± 4.05	56.47 ± 2.47
Normal morphology %	4.00 ± 0.20	3.63 ± 0.12	3.47 ± 0.16	3.63 ± 0.09
TMS %	50.42 ± 3.20	44.82 ± 1.64	44.45 ± 2.08	45.46 ± 1.19
Viability (HOS) %	73.32 ± 2.47	70.13 ± 1.28	70.49 ± 1.57	70.70 ± 0.92
Oxidant/antioxidant concentrations				
ROS (U/min)	1.60 ± 0.12	1.71 ± 0.07	1.72 ± 0.08	1.70 ± 0.05
SOD (U/min)	13.73 ± 0.35	13.42 ± 0.17	13.47 ± 0.20	13.48 ± 0.12
GPX (nmol)	10.84 ± 0.08	10.63 ± 0.05	10.66 ± 0.05	10.67 ± 0.03
CAT (g/dl)	9.71 ± 0.14	9.62 ± 0.06	9.62 ± 0.09	9.63 ± 0.05
MDA (nmol/ml)	28.56 ± 0.24	28.82 ± 0.11	28.86 ± 0.18	28.80 ± 0.09
Reproductive hormone levels				
FSH (mIU/ml)	6.08 ± 0.40	5.50 ± 0.19	5.91 ± 0.35	5.71 ± 0.16
LH (mIU/ml)	8.10 ± 1.35	6.75 ± 0.52	8.67 ± 1.00	7.54 ± 0.46
Prolactin (mIU/ml)	10.84 ± 1.12	11.34 ± 0.52	13.13 ± 0.72	11.84 ± 0.40
Testosterone (ng/ml)	382.25 ± 31.82	365.55 ± 15.63	394.80 ± 19.55	377.18 ± 11.40

Values represent mean ± SEM; n = number of patients; WBC, white blood cell; TMS, total motile sperm; HOS, hypo-osmotic swelling, ROS, reactive oxygen species; SOD, superoxide dismutase; GPX, guaiacol peroxidase; CAT, catalase; MDA, lipid peroxidation; FSH, follicular stimulating hormone; LH, luteinizing hormone.

### 3.3 Sperm chromatin integrity parameters

**Figure 1:**
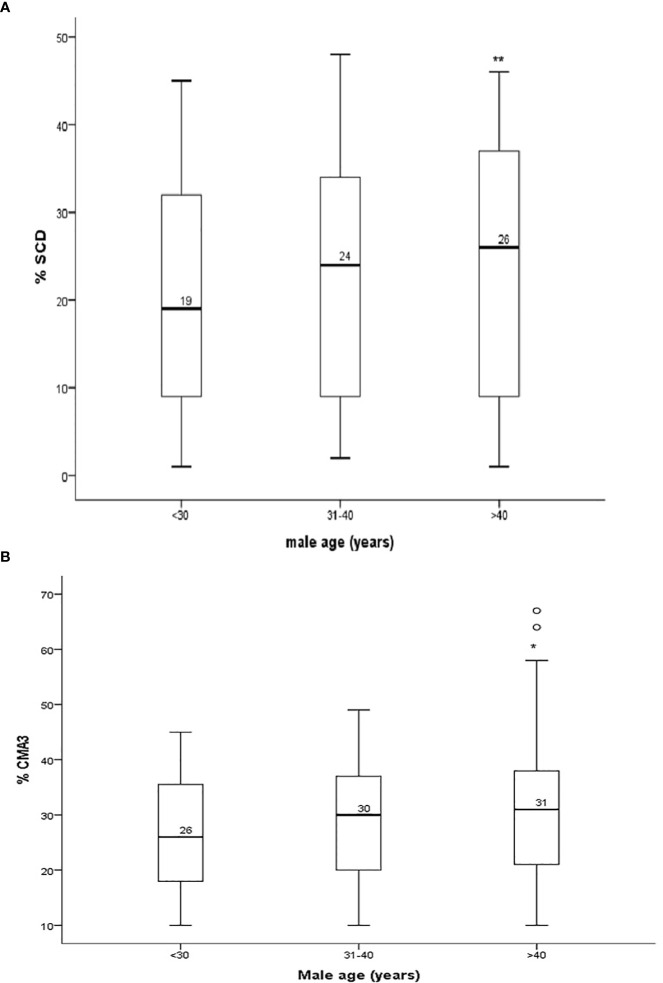
**(A)** Percentage of sperm chromatin dispersion (SCD) and **(B)** sperm protamine (CMA3+) content in sperm of males in different age groups. *P < 0.05, **P < 0.01.

**Figure 2:**
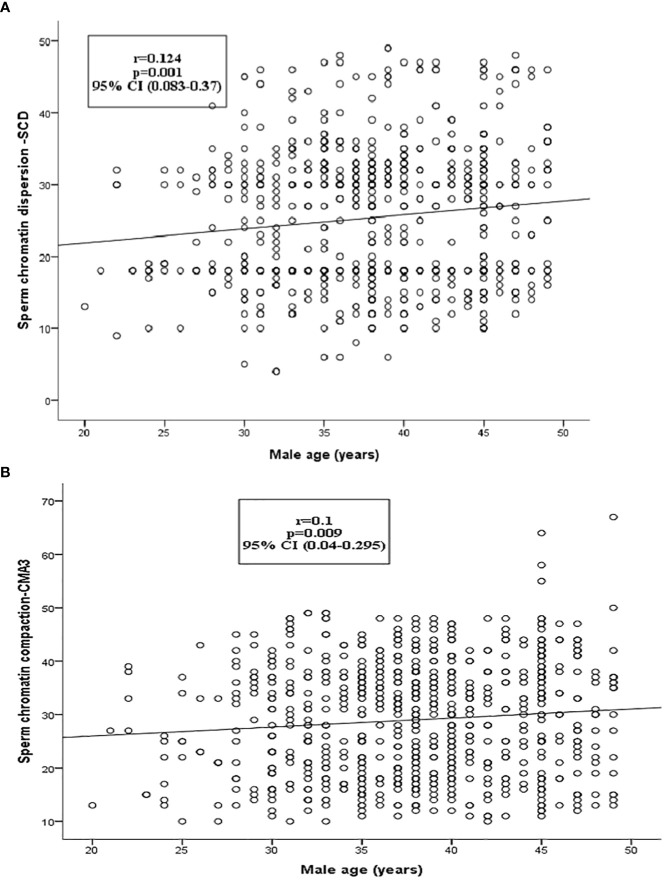
The relationship between sperm chromatin dispersion and sperm chromatin compaction (CMA3+) to male age. **(A)** The left charts show scatterplot correlation lines depicting the association between sperm chromatin dispersion (SCD) and male age. **(B)** Sperm chromatin compaction/protamine (CMA3+) and male age.

We found that aged men (>40 years) had a higher percentage of sperm with DNA damage (26.6 ± 0.6, *p* = 0.001) compared to younger aged men (¾30 years age, SCD% = 23.2 ± 0.88) (**Table 3**, **Figure 1A**). Percentage of mature spermatozoa with intact chromatin (CMA3) significantly (*p* = 0.04) decreased with the age of men (**Table 3**, **Figure 1B**). A significant positive correlation was found between the age of men and percentage of spermatozoa with DNA damage (SCD) (*r* = 0.124, *p* = 0.001) and percentage of immature spermatozoa with abnormal chromatin compaction (CMA3) (*r* = 0.1, *p* = 0.009) (**Figures 2A, B**). A significant positive linear association was found between male age and spermatozoa abnormal chromatin compaction (CMA3%) [*β* = 0.169, *t* = 2.63, 95% CI (0.042–0.295); *p* = 0.009] and spermatozoa with higher percentage of fragmented DNA (SCD %) [*β* = 0.195, *t* = 3.42, 95% CI (0.08–0.307); *p* = 0.001].

**Table 3:** Male age influence sperm chromatin dispersion (SCD), chromatin integrity (TB+), and chromatin compaction (CMA3+) in studied groups.

	Below 30 years (*n* = 90)	30 to 40 years (*n* = 330)	Above 40 years (*n* = 330)	Total (*n* = 750)
Sperm chromatin dispersion—SCD %	23.2 ± 0.88	25.1 ± 0.4	26.6 ± 0.6**	25.4 ± 0.34
Chromatin integrity—TB+ %	26.71 ± 1.83	26.47 ± 0.86	28.65 ± 1.14	27.23 ± 0.65
Chromatin compaction—CMA3+ %	26.6 ± 1.03	29.04 ± 0.50	30 ± 0.71*	28.9 ± 0.30

Values represent mean ± SEM; n = number of patients; SCD, sperm chromatin dispersion; TB+, toluidine blue staining; CMA3+, chromomycin A3 staining.

*p < 0.05, **p < 0.01.

## 4 Discussion

The results of this study confirmed that advancing male age is associated with impaired sperm quality and sperm chromatin integrity. In the current investigation, we found an association between sperm DNA damage and rising male age. The alteration of sperm compactness (CMA3) in early stages of spermatogenesis leads to sperm DNA damage. Higher sperm DNA damage percentage was directly linked to increased male age. Male age harmed the integrity of sperm chromatin and its condensation, which represents a higher percentage of immature sperm with less compact chromatin (CMA3) (39, 40). It has been reported that, with age, ejaculated spermatozoa do exhibit changes, consistent with apoptosis in somatic cells, such as external of phosphatidylserine (PS), disrupted mitochondrial membrane potential, and/or DNA fragmentation (41). Recently, apoptosis has received much attention because of its vital role in reproduction, and early apoptosis indicated as the percentage of spermatozoa with PS, which is normally sequestered in the plasma membrane inner leaflet and appears in the outer leaflet, triggers non-inflammatory phagocytic reaction. Despite the effectiveness of DNA repair mechanisms, some DNA damage goes unrepaired, resulting in a gradual accumulation of DNA lesions in cells with mature age. As a result, the gradual but steady accumulation of damaged cells within tissues occurs with human aging (42). In the current investigation, we found an association between sperm DNA damage and rising male age (SCD); the present findings are consistent with other studies (43–45) in that higher sperm DNA damage percentage was directly associated with increased male age (46). A study showed a positive relationship between male age and sperm DNA damage in oligoasthenoteratozoospermia (OAT) but no difference in the control group (47), while some studies did not find any change in sperm DNA damage with an increase in male age (48–50). Male age harmed the integrity of sperm chromatin and its condensation, which represents a higher percentage of immature sperm CMA3.

In the current study, advanced men’s age causes an increased risk of sperm chromatin de-condensation compared to younger men. The decrease in protamination, or possibly an issue with protamines caused by reduced thiol levels, would most likely explain the rise in CMA3+ staining. This would increase the histone-to-protamine ratio, which is what causes male subfertility (51). Alternate hypotheses for the etiology include immature spermatozoa shedding from the seminiferous tubes and abnormal protamine dephosphorylation (40, 52, 53). There was very limited literature on the influence of advanced male age on sperm chromatin packaging in humans and on data suggesting advanced human male age to be related to higher sperm chromatin damage (54).

Our analyses found no influence of male age on sperm morphology, motility, and concentration. Moreover, we looked into the relationship between male age and oxidative stress levels. A previous study showed a strong correlation between sperm DNA fragmentation and poor sperm quality, although no preferential effect on sperm concentration or morphology seemed to be present (55, 56). ROS production and levels of antioxidant enzyme imbalance result in impaired male fertility potential, and there are contradictory results on the relationship between levels of ROS production in semen with advanced male age (23, 49, 57–60). One study found a positive relationship (61), while another found no relationship between male age and ROS higher production. The present study found no link between male age with ROS production and no difference in ROS levels and antioxidative agents in all age groups (62). Given that the OS is a major factor affecting sperm function and that the balance between pro- and antioxidative agents is frequently shifted towards the pro-oxidizing condition in aging testis mitochondria, antioxidant interventions hold great promise as therapeutic strategies to lessen the negative effects of aging (and the resulting oxidative stress) on the male reproductive system (63, 64). The analysis of the present study revealed that there was no association between male age and reproductive hormone concentration. Androgen hormones are linked directly to sperm quality parameters and reproductive hormone imbalance leads to impaired spermatogenesis and poor male sexual health (65, 66). Male aging has previously been linked to a variety of factors, including decreased sperm quality, hormonal imbalances, and longer pregnancy times. Recent data, however, indicate that healthy aging does not impair spermatogenic output or hormone production from the testicles (1, 67).

As a result, we may also draw the additional conclusion that having older fathers has a deleterious effect on the molecular makeup of motile spermatozoa (23, 68). Given that it is established that sperm DNA is well protected because of chromatin condensation, which is essential at the time of sperm transit in the female reproductive system and additionally to manipulate epigenetic reprogramming at some point during the pre-implantation period, an increase in male age could result in impaired sperm chromatin integrity, making spermatozoa’s genetic material vulnerable to the external environment insult (64, 69). Similar to this, poor fertility outcomes such as low fertilization rates, embryo morphokinetics, recurrent implantation failures, and miscarriages are associated with chromatin condensation and DNA integrity (67, 70–72). The process of sperm genome modification is believed to be due to highly hierarchical epigenetic changes occurring in the paternal genome after fertilization, including the dissolution of the sperm nuclear envelope, decondensation of the genetic material *via* the breakage of the disulfide bridges among protamines, substitution of maternal histones for male protamines, and genetic material rearrangement (64, 73). Understanding the body of available scientific evidence is the first step toward reducing or mitigating the negative effects of advanced male age. The present study sheds new light on the intricate associations between male age and concentrations of FSH and LH as well as DNA fragmentation and chromatin deficiency of spermatozoa among healthy men of reproductive age undergoing ICSI treatment. However, the results from this study should help provide critical information to assisted reproduction physicians and clinicians to understand the risks associated with male age and the resulting progenies after IVF/ICSI treatment. In the field of assisted reproduction, our study suggests that older men who are seeking fertility treatment may require more extensive testing and treatment than younger men. It also highlights the importance of seeking fertility treatment in younger age in male subjects; as the male age advances, fertility potential is reduced. Furthermore, the population in this study was homogeneous. Researchers, healthcare professionals, decision-makers, and patients, among others, should continue to discuss new data and their implications for individuals and society. Above all, it is critical that all parties work together to create a new agenda for reconsidering advanced male age management strategies in the context of protecting future parents’ reproductive health.

The disadvantage of this study is that the sample size in Group 1 was smaller as compared to the rest, which is due to the recent social changes that enable men and women to choose to have a career first and delay childbearing and fatherhood to later age. The content and extent of fatherhood duties are filled in by traditional gender roles mainly set by society. The father provides protection and income for the mother and child. Financial and professional security and a greater motivation for parenthood usually characterized older couples. Moreover, the absence of an explicit condemnation of the fatherhood age of men encourages a large number of men to delay fatherhood to advanced age. Secondly, it does not take into account other confounding factors, such as family histories and other diseases of old age. Subsequent cohort studies with older and younger men undergoing assisted reproductive treatment are recommended to investigate the effects of male advancing age on sperm chromatin packaging.

Male age identified by our investigation is an independent risk component for sperm DNA damage and chromatin condensation and influences reproductive health that could alter pre- and post-embryological developmental stages. This finding needs to be confirmed by future large prospective studies.

## 5 Conclusion

Old-aged men had a higher percentage of spermatozoa with sperm DNA damage (SCD %), significantly higher levels of immaturity (chromomycin staining, CMA3%), and a lower level of chromatin integrity. Male age is one of the factors contributing to the decline of male fertility. Therefore, younger age is advisable for patients who are undergoing assisted reproductive therapy.

## Data availability statement

The raw data supporting the conclusions of this article will be made available by the authors, without undue reservation.

## Ethics statement

The ethical board of Quaid-i-Azam University and SKMC Islamabad Pakistan approved the experimental protocol # BEC-FBS-QAU2016-77 for the use of humans in this work. Participants were asked for their written consent. The patients/participants provided their written informed consent to participate in this study.

## Author contributions

RB, SJ, SR, SQ, TA, AA, MQ, MH and HA significantly contributed to the design, experimental design, data collection, statistical evaluation, and article writing. RB, SJ, SQ, TA, SR, AA, MQ, MH and HA all contributed significantly to data interpretation and manuscript revision for intellectual content. All authors contributed to the article and approved the submitted version.
